# Reliability of Point-of-Care Vitamin D Testing for Use in Primary Health Care: A Comparative Evaluation with Established CLIA Platforms

**DOI:** 10.2147/IJGM.S596577

**Published:** 2026-07-24

**Authors:** Salma Younes, Tasneem AlHamad, Alma Al-louzi, Fatima Al Khanji, Rahma Mohamed Ibrahim, Dania Yaseen, Reem Al-Ansari, Asmaa Eltaweel, Farah Trad, Parveen B Nizamuddin, Nouran Zein, Nadin Younes, Dayana El Chaar, Patrick Tang, Nader Al-Dewik, Laith J Abu-Raddad, Gheyath K Nasrallah

**Affiliations:** 1 Biomedical Sciences Department, College of Health Sciences, Qatar University, Doha, Qatar; 2 Biomedical Research Center, Qatar University, Doha, Qatar; 3 Department of Pathology, Sidra Medicine, Doha, Qatar; 4 Department of Research, Women’s Wellness and Research Center, Hamad Medical Corporation, Doha, Qatar; 5 Infectious Disease Epidemiology Group, Weill Cornell Medicine–Qatar, Cornell University, Qatar Foundation, Education City, Doha, Qatar; 6 World Health Organization Collaborating Centre for Disease Epidemiology Analytics on HIV/AIDS, Sexually Transmitted Infections, and Viral Hepatitis, Weill Cornell Medicine–Qatar, Cornell University, Qatar Foundation, Education City, Doha, Qatar; 7 Department of Healthcare Policy and Research, Weill Cornell Medicine, Cornell University, New York, NY, USA

**Keywords:** vitamin D, 25-hydroxyvitamin D, chemiluminescent immunoassay, point-of-care testing, analytical performance, diagnostic agreement

## Abstract

**Background:**

Accurate measurement of serum 25-hydroxyvitamin D [25(OH)D] is essential for diagnosing deficiency and guiding treatment. While automated chemiluminescent immunoassays (CLIAs) are widely used in centralized laboratories, growing demand for rapid decisions has increased interest in reliable point-of-care (POC) testing. However, comparative data between modern POC systems and established laboratory analyzers remain limited.

**Aim:**

To assess the analytical agreement and diagnostic performance of a fluorescence-based POC vitamin D immunoassay and a contemporary automated CLIA platform against a benchmark automated CLIA system.

**Methods:**

A total of 311 human serum samples covering deficient, insufficient, sufficient, and high 25(OH)D concentrations were analyzed using one fluorescence-based POC assay and two automated CLIA platforms. Agreement was evaluated using Spearman correlation, Bland–Altman analysis, and receiver operating characteristic (ROC) curves. Diagnostic performance for vitamin D insufficiency (<30 ng/mL) was assessed by sensitivity, specificity, positive predictive value (PPV), negative predictive value (NPV), overall percent agreement (OPA), and Cohen’s κ.

**Results:**

All three assays showed excellent concordance, with strong correlations (r = 0.86–0.92) and similar median 25(OH)D concentrations. Bland–Altman analysis demonstrated modest, clinically acceptable biases (–14.3% to +10.0%) without proportional error. Diagnostic agreement was high, with AUC values of 0.96–0.98 and Cohen’s κ values of 0.84–0.85, indicating high agreement in classifying vitamin D insufficiency. The fluorescence-based POC assay showed performance comparable to both automated CLIAs, with specificity of 91–96% and NPV of 91–97%.

**Conclusion:**

Both the fluorescence-based POC immunoassay and the contemporary automated CLIA platform demonstrated high diagnostic agreement with the benchmark automated CLIA system. These findings support the potential utility of both laboratory-based and decentralized assays for routine vitamin D screening in appropriate clinical settings.

## Introduction

Vitamin D (VitD) is essential for maintaining bone integrity, supporting immune function, and regulating metabolic processes.^1–3^ However, VitD deficiency remains a significant global health issue, impacting individuals across all ages and geographic regions.^4^ In Qatar, vitamin D deficiency remains highly prevalent at the population level, underscoring the clinical importance of reliable 25(OH)D testing in routine care.^5^ Low serum levels of 25-hydroxyvitamin D [25(OH)D], the key biomarker for assessing VitD status, are linked to skeletal conditions like rickets and osteomalacia, as well as an elevated risk of autoimmune disorders, cardiovascular diseases, and metabolic syndromes.^1–3^ Accurate and timely identification of VitD deficiency is critical for its management and prevention.

Automated chemiluminescent immunoassays (CLIAs) are widely adopted in routine laboratories because they combine throughput, automation, and operational efficiency. Certain automated CLIA platforms have been extensively used in clinical practice and are frequently treated as benchmark systems for 25(OH)D testing.^6^ However, liquid chromatography–tandem mass spectrometry (LC-MS/MS) remains the recognized reference method for absolute vitamin D quantification and assay standardization initiatives such as VDSP and DEQAS. Newer technologies, including high-throughput automated CLIA analyzers and fluorescence-based point-of-care (POC) immunoassays, aim to deliver comparable analytical performance while expanding access through streamlined workflows and decentralized, near-patient testing. Nevertheless, inter-assay variability, differences in antibody specificity, calibration traceability, and potential matrix effects can complicate result comparability and clinical interpretation across platforms.^7–11^

Only a limited number of studies have evaluated POC platforms for vitamin D assessment, including lateral flow fluorescence immunoassays, cartridge-based immunoassays, and portable analyzers designed for near-patient testing. Although these systems offer faster turnaround times and improved accessibility, the few available comparative studies remain limited by relatively small sample sizes, restricted concentration ranges, or limited representation of clinically relevant vitamin D categories.^12^,^13^ Therefore, additional validation studies are needed, particularly using serum samples spanning deficient, insufficient, sufficient, and high 25(OH)D concentrations.

In this study, we compare a contemporary automated CLIA analyzer and a fluorescence-based POC immunoassay directly against an established benchmark automated CLIA platform using a clinically diverse cohort. We quantify inter-assay relationships via correlation and Bland–Altman analyses and evaluate diagnostic performance at clinically relevant thresholds using sensitivity, specificity, predictive values, overall percent agreement (OPA), and Cohen’s κ. This design provides practice-oriented evidence on whether newer laboratory-based and near-patient modalities can be used interchangeably with established systems for routine assessment of vitamin D status.

## Methods

### Sample Collection and Ethical Approval

For this study, 311 serum samples were sourced from the Qatar Biobank. To maintain sample integrity and prevent degradation from repeated freeze-thaw cycles, the samples were aliquoted and stored at −80°C. The cohort was meticulously curated to include a wide spectrum of vitamin D levels, ranging from deficiency and insufficiency to sufficiency and toxicity. The cohort included samples from individuals with conditions commonly encountered in routine vitamin D testing, including pregnancy, chronic kidney disease, and osteoporosis. These samples were intentionally included to better reflect real-world clinical testing conditions rather than limiting the evaluation to healthy individuals. However, detailed subgroup-level stratified analyses were beyond the scope of the present study and should be evaluated in future investigations.

As this study was designed as a retrospective comparative assay evaluation using available serum samples from Qatar Biobank, a formal a priori power calculation was not performed. However, the final cohort included 311 serum specimens, exceeding the commonly recommended minimum of 40 patient samples for method-comparison studies described in CLSI EP09c. The cohort was selected to cover a broad clinical range of 25(OH)D concentrations, including deficient, insufficient, sufficient, and high categories, allowing assessment of inter-assay agreement across clinically relevant decision ranges.^14^

Ethical approval for the study was obtained from the Institutional Review Board at Qatar University (IRB# QU-IRB 2011-E/23), ensuring adherence to rigorous ethical standards and the confidentiality of participant data. The study was conducted in accordance with the principles of the Declaration of Helsinki, with full protection of participant confidentiality and adherence to institutional ethical standards.

### Diagnostic Assay Methodologies

Serum 25-hydroxyvitamin D [25(OH)D] concentrations were measured using three analytical platforms: one fluorescence-based point-of-care (POC) immunoassay and two automated chemiluminescent immunoassay (CLIA) systems (Table 1).

**Table 1:** Technical Comparison of the Three Vitamin D Assays

Parameter	DiaSorin LIAISON^®^	Mindray CL-900i	Wondfo Finecare
Methodology	CLIA	CLIA	Fluorescence immunoassay (FIA)
Assay principle	Direct competitive chemiluminescent immunoassay	Competitive chemiluminescent immunoassay	Fluorescent competitive immunoassay
Detection signal	Relative light units (RLUs), inversely proportional to 25(OH)D concentration	Relative light units (RLUs)	Fluorescence signal
Setting	Central laboratory	Central laboratory	Point-of-care
Sample type	Serum	Serum	Whole blood, serum, plasma
Measurement range/sensitivity	4–150 ng/mL	3.0–150 ng/mL	5–100 ng/mL
Principle details	25(OH)D dissociated from binding protein, binds to solid-phase antibody, followed by isoluminol-labeled vitamin D tracer	Competitive CLIA principle	Fluorescent competitive assay
Turnaround time/workflow	Automated laboratory assay	Automated laboratory assay	Rapid (~10 min)

The automated CLIA platforms included a high-throughput analyzer (CL-900i^®^, Mindray Bio-Medical Electronics, China) and a benchmark automated system (LIAISON^®^, DiaSorin, Italy). The POC platform consisted of a fluorescence immunoassay-based analyzer (Finecare™, Guangzhou Wondfo Biotech, China). Each platform operates on distinct analytical principles, allowing comprehensive evaluation of assay performance, precision, and diagnostic reliability across technological categories.

#### Automated CLIA Analyzer (High-Throughput Platform)

Serological testing was performed using an automated chemiluminescent immunoassay analyzer for quantitative detection of serum 25(OH)D. The assay is based on a competitive chemiluminescence immunoassay principle. Following incubation, the chemiluminescent reaction is measured as relative light units (RLUs) by an integrated photomultiplier system. According to the manufacturer’s specifications, serum 25(OH)D concentrations <30 ng/mL are classified as insufficient, while values between 30–100 ng/mL are considered sufficient.^15^

#### Benchmark Automated CLIA Platform

The benchmark automated CLIA system operates on a direct competitive immunoassay principle. During initial incubation, 25(OH)D is released from its binding proteins and binds to specific antibodies immobilized on a solid phase. After incubation with a chemiluminescent tracer, emitted light is measured in relative light units (RLUs), which inversely correlate with serum 25(OH)D concentrations.

#### Fluorescence-Based Point-of-Care Immunoassay

The fluorescence-based POC vitamin D assay is designed for quantitative determination of total 25(OH)D (25(OH)D_2_ + 25(OH)D_3_) in human serum or plasma. The assay utilizes a competitive fluorescence immunodetection method in which 25(OH)D in the sample competes with a fluorescently labeled tracer for binding to specific antibodies. After incubation, unbound tracer migrates along a test strip and binds to immobilized analyte, generating a fluorescence signal inversely proportional to 25(OH)D concentration. The signal is read by a dedicated fluorescence immunoassay meter, which reports results in ng/mL. According to manufacturer specifications, the analytical measurement range is 5–100 ng/mL, with values below 5 ng/mL reported as “<5 ng/mL” and above 100 ng/mL as “>100 ng/mL.” Reported intra-assay precision is ≤10% coefficient of variation (CV), and inter-assay precision is ≤15%. Manufacturer validation studies indicate strong correlation with established laboratory immunoassays.

### Statistical Analysis

All statistical analyses were performed using GraphPad Prism version 9.3.1 (San Diego, CA, USA). The Shapiro–Wilk test assessed data normality. As data were not normally distributed, nonparametric analyses, including the Kruskal–Wallis test, were applied. Numerical data are presented as medians and interquartile ranges (IQR), with p < 0.05 considered statistically significant.

Diagnostic performance metrics—including sensitivity, specificity, positive predictive value (PPV), negative predictive value (NPV), and overall percent agreement (OPA)—were calculated with 95% confidence intervals (CIs). Clinical categories were defined according to the 2011 Endocrine Society Clinical Practice Guideline as follows: deficiency (<20 ng/mL), insufficiency (20–29 ng/mL), sufficiency (30–100 ng/mL), and potential toxicity (>100 ng/mL). For ROC analysis and diagnostic performance calculations, a binary clinical threshold of <30 ng/mL was used, whereby samples with 25(OH)D concentrations <30 ng/mL were classified as deficient/insufficient and samples ≥30 ng/mL were classified as sufficient. These thresholds were used for categorical assay-comparison purposes, while acknowledging that vitamin D cutoffs remain debated and that alternative frameworks, such as the Institute of Medicine/National Academy of Medicine (IOM/NAM), consider approximately ≥20 ng/mL sufficient for bone health in most individuals.^16^

Agreement between assays was evaluated using Cohen’s κ coefficient (<0.40 poor; 0.40–0.59 fair; 0.60–0.74 good; ≥0.75 excellent). Bland–Altman plots assessed concordance and systematic bias. Spearman’s rank correlation coefficients were used to assess monotonic relationships between assays; categorized as very weak (0.00–0.19), weak (0.20–0.39), moderate (0.40–0.59), strong (0.60–0.79), and very strong (0.80–1.00). Linear regression lines with 95% confidence intervals were added to scatterplots for visualization purposes only and were not used for inferential statistical analysis.

Receiver operating characteristic (ROC) curves were generated to assess diagnostic discrimination, and the area under the curve (AUC) was calculated. AUC values were interpreted as follows: 0.9–1.0 excellent; 0.8–0.9 very good; 0.7–0.8 good; 0.6–0.7 sufficient; 0.5–0.6 poor; <0.5 not useful.^17^

ROC analyses were performed using pairwise comparisons between assays rather than against a reference gold standard. The DiaSorin LIAISON platform was selected as the benchmark comparator due to its widespread clinical use and extensive validation in vitamin D testing literature.

## Results

### Distribution of VitD Levels Across Assays

**Figure 1:**
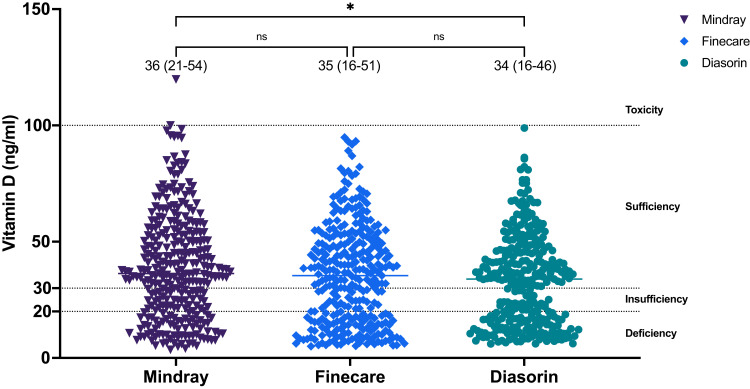
Distribution of serum 25-hydroxyvitamin D [25(OH)D] concentrations across the three assay platforms. Each point represents an individual serum sample (n = 311) measured using the contemporary automated CLIA platform (Mindray CL-900i), fluorescence-based point-of-care (POC) immunoassay (Finecare), and benchmark automated CLIA platform (DiaSorin LIAISON). Horizontal bars indicate median values, with interquartile ranges (IQR) shown above each group. Group differences were assessed using the Kruskal–Wallis test with Dunn’s post hoc correction (* = *p* < 0.05, ns = not significant). Horizontal dotted lines indicate clinical thresholds for deficiency (<20 ng/mL), insufficiency (20–30 ng/mL), sufficiency (30–100 ng/mL), and toxicity (>100 ng/mL).

The distribution of vitamin D concentrations showed overall consistency across the three evaluated platforms—two automated CLIA systems and one fluorescence-based point-of-care assay—with minor variations in central tendency (Figure 1). The high-throughput automated CLIA analyzer recorded the highest median 25(OH)D concentration at 36 ng/mL (IQR: 21–54), followed by the point-of-care assay at 35 ng/mL (IQR: 16–51) and the benchmark automated CLIA system at 34 ng/mL (IQR: 16–46). Kruskal–Wallis testing revealed a statistically significant overall difference among the three platforms (p < 0.05). Post-hoc pairwise comparisons showed that the high-throughput CLIA analyzer reported slightly higher values than the benchmark CLIA system (p < 0.05), whereas differences between the point-of-care assay and the two CLIA systems were not statistically significant (p ≥ 0.05). Across all assays, most samples fell within the sufficiency range (20–100 ng/mL), with relatively few values in the deficiency range (<20 ng/mL) and only isolated measurements exceeding the toxicity threshold (>100 ng/mL). These findings indicate broadly comparable classification of vitamin D status at the population level, with the high-throughput CLIA platform exhibiting a modest positive bias relative to the benchmark system.

### Correlation Analysis

**Figure 2:**
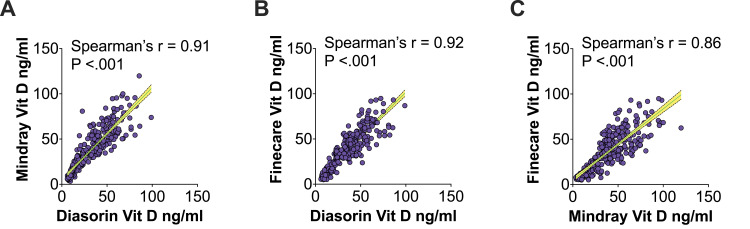
Pairwise Spearman correlation analysis of serum 25-hydroxyvitamin D [25(OH)D; abbreviated as Vit D on the axes] concentrations among the three assay platforms. Scatterplots show pairwise comparisons between the contemporary automated CLIA platform, fluorescence-based POC immunoassay, and benchmark automated CLIA platform. Spearman correlation coefficients (r) and corresponding p-values are displayed in each panel. Fitted trend lines were added only to illustrate overall measurement alignment and were not used as the primary statistical model. (**A**) Mindray vs DiaSorin; (**B**) Finecare vs DiaSorin; (**C**) Finecare vs Mindray.

Spearman’s rank correlation analysis (Figure 2) demonstrated very strong and statistically significant correlations among the three evaluated assays, with correlation coefficients ranging from r = 0.86 to 0.92 (p < 0.001). The strongest association was observed between the point-of-care assay and the benchmark automated CLIA system (r = 0.92), followed closely by the two automated CLIA platforms (r = 0.91). The correlation between the point-of-care assay and the high-throughput CLIA analyzer (r = 0.86), although slightly lower, remained within the very strong range. Overall, these findings confirm that the three immunoassay-based platforms exhibit high internal consistency across the full clinical range of 25(OH)D concentrations, supporting their interchangeability for routine clinical assessment.

### Agreement Analysis

**Figure 3:**
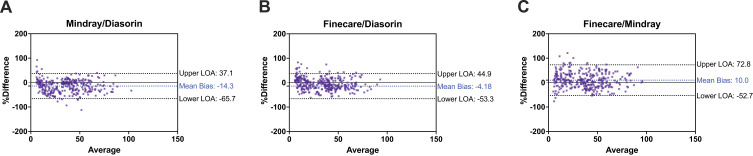
Bland–Altman agreement analysis of serum 25(OH)D measurements across the three assay platforms. Pairwise comparisons include (**A**) contemporary automated CLIA vs benchmark automated CLIA, (**B**) fluorescence-based POC immunoassay vs benchmark automated CLIA, and (**C**) fluorescence-based POC immunoassay vs contemporary automated CLIA. The y-axis represents percentage difference between paired measurements, while the x-axis shows the average 25(OH)D concentration (ng/mL). The blue horizontal line indicates mean bias, and black dotted lines represent the 95% limits of agreement (LOA). Mean bias and LOA values are annotated within each panel.

Bland–Altman analysis demonstrated consistent agreement among the three immunoassays, with mean percentage biases ranging from –14.3% to +10.0% and no evidence of proportional error (Figure 3). When the benchmark automated CLIA system was used as the common reference, both the high-throughput CLIA analyzer and the fluorescence-based point-of-care assay showed predictable and clinically acceptable offsets. The high-throughput CLIA analyzer exhibited a mean bias of –14.3%, indicating that the benchmark system systematically reported slightly lower 25(OH)D values across the measurement range. The point-of-care assay demonstrated a smaller negative bias (–4.18%) relative to the benchmark system, again reflecting marginally lower values from the reference platform with narrow limits of agreement. When the point-of-care assay was compared directly with the high-throughput CLIA analyzer, the mean bias shifted to +10.0%, indicating modestly higher values from the POC platform, although dispersion remained symmetrical and within clinically acceptable limits. Collectively, these findings confirm strong analytical coherence across all platforms. The observed systematic differences were small, clinically manageable, although acceptable at the population level, such biases may influence classification outcomes in individual patients with values near clinical decision thresholds.

### ROC Analysis

**Figure 4:**
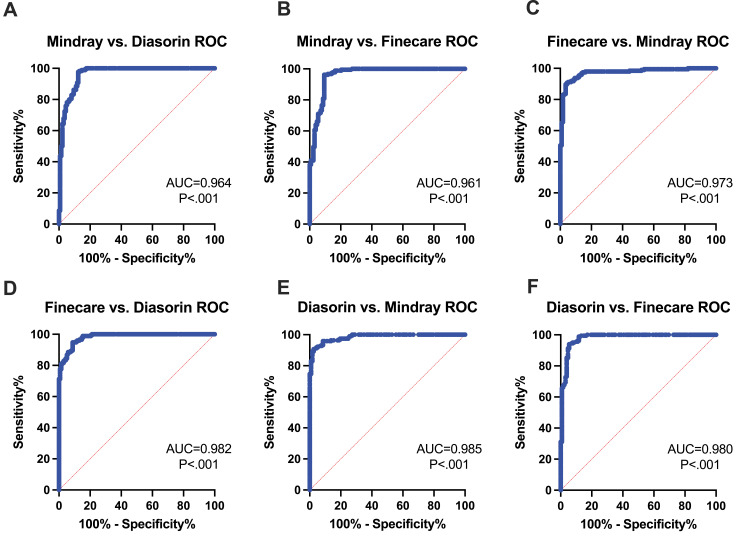
Receiver operating characteristic (ROC) analysis of diagnostic agreement among the three assay platforms for classification of vitamin D insufficiency. Binary classification was based on serum 25(OH)D concentrations <30 ng/mL (insufficiency) versus ≥30 ng/mL (sufficiency). Panels show pairwise comparisons between the contemporary automated CLIA platform, fluorescence-based POC immunoassay, and benchmark automated CLIA platform, with sensitivity plotted against 1 − specificity. Area under the curve (AUC) values and *p*-values are displayed in each panel (all *p* < 0.001). The red diagonal line represents the line of no discrimination (AUC = 0.5). (**A**) Mindray vs DiaSorin; (**B**) Mindray vs Finecare; (**C**) Finecare vs Mindray; (**D**) Finecare vs DiaSorin; (**E**) DiaSorin vs Mindray; (**F**) DiaSorin vs Finecare.

Receiver operating characteristic (ROC) analysis (Figure 4) was performed to evaluate diagnostic agreement among the three immunoassay platforms using the clinical cutoff of <30 ng/mL to define vitamin D insufficiency. All pairwise comparisons demonstrated excellent discrimination, with AUC values ranging from 0.961 to 0.985 (all p < 0.001). The highest concordance was observed between the two automated CLIA systems (AUC = 0.964), reflecting strong diagnostic alignment between laboratory-based platforms. The fluorescence-based point-of-care assay also demonstrated excellent performance, with AUC values of 0.982 when compared with the benchmark automated CLIA system and 0.973 when compared with the high-throughput CLIA analyzer. Overall, these findings confirm that all three immunoassays exhibit near-perfect ability to classify vitamin D insufficiency, supporting their diagnostic consistency across laboratory and decentralized testing settings.

### Diagnostic Performance Metrics

Diagnostic accuracy was assessed through pairwise comparisons among the three immunoassay platforms using sensitivity, specificity, positive predictive value (PPV), negative predictive value (NPV), overall percent agreement (OPA), and Cohen’s κ (Table 2). These metrics evaluate each assay’s ability to consistently classify individuals as vitamin D insufficient (<30 ng/mL) or sufficient (≥30 ng/mL). Across all comparisons, the three platforms demonstrated uniformly high diagnostic concordance. Agreement between the two automated CLIA systems yielded a sensitivity of 84%, specificity of 99%, OPA of 92%, and κ = 0.84, indicating strong classification consistency between laboratory-based analyzers. The fluorescence-based point-of-care assay also showed high agreement with both CLIA platforms. When compared with the high-throughput automated analyzer, sensitivity was 95%, specificity 91%, OPA 93%, and κ = 0.85. When compared with the benchmark automated CLIA system, sensitivity was 88%, specificity 96%, OPA 93%, and κ = 0.85. Reverse comparisons produced nearly identical performance estimates, confirming strong internal consistency across all three immunoassays. Overall, the diagnostic metrics indicate comparable accuracy in classifying vitamin D insufficiency, supporting practical interchangeability in routine clinical assessment.

**Table 2:** Summary of the Diagnostic Performance of the Three CLIA

TestxReference	K	OPA %	PPV %	NPV %	Sensitivity	Specificity
Contemporary CLIA x Benchmark CLIA	0.84 (0.78–0.9)	92 (89–95)	98 (94–100)	89 (83–93)	84 (77–90)	99 (96–100)
Contemporary CLIA × Fluorescence-Based POC	0.85 (0.78–0.91)	93 (89–95)	95 (89–98)	91 (86–95)	87 (80–92)	97 (93–99)
Fluorescence-Based POC x Contemporary CLIA	0.85 (0.78–0.91)	93 (89–95)	87 (80–92)	97 (93–99)	95 (89–98)	91 (86–95)
Fluorescence-Based POC x Benchmark CLIA	0.85 (0.79–0.91)	93 (89–95)	95 (89–98)	91 (86–95)	88 (82–93)	96 (92–98)
Benchmark CLIA × Contemporary CLIA	0.84 (0.78–0.9)	92 (89–95)	84 (77–90)	99 (96–100)	98 (94–100)	89 (83–93)
Benchmark CLIA × Fluorescence-Based POC	0.85 (0.79–0.91)	93 (89–95)	88 (82–93)	96 (92–98)	95 (89–98)	91 (86–95)

## Discussion

Accurate measurement of 25-hydroxyvitamin D [25(OH)D] remains central to diagnosing deficiency and guiding supplementation strategies in populations at risk of bone and metabolic disorders.^1^,^2^,^4^,^18^,^19^ Despite technological advances, inter-method variability continues to limit result comparability and clinical decision-making.^10^,^11^,^16^ In this context, the present study provides a structured comparative evaluation of three immunoassay-based diagnostic platforms, one benchmark automated chemiluminescent immunoassay (CLIA), one contemporary automated CLIA, and one fluorescence-based point-of-care (POC) immunoassay, within a clinically diverse cohort. By integrating distributional, correlation, agreement, and diagnostic performance analyses, the findings clarify the degree of analytical concordance and practical interchangeability among laboratory-based and near-patient testing modalities.

Across all analytical assessments, the three immunoassays demonstrated strong internal agreement and closely aligned central tendencies. Median 25(OH)D concentrations differed by fewer than 5 ng/mL across platforms, indicating minimal population-level systematic offset (Figure 1). Although the contemporary CLIA platform demonstrated statistically higher median values than the benchmark platform, the absolute difference was small (<5 ng/mL) and remained within clinically acceptable limits. This discrepancy likely reflects minor calibration differences, antibody specificity variation, or manufacturer-specific standardization approaches rather than clinically meaningful disagreement. The observed Bland–Altman mean biases were within the broader College of American Pathologists (CAP) Accuracy-Based Vitamin D Survey acceptability criterion for total 25-OH vitamin D, defined as results within ±25% of the CDC reference value or ±5 ng/mL, whichever is greater. However, these findings should not be interpreted as formal CDC/VDSP standardization or certification, as this requires comparison with a reference measurement procedure and assessment of both bias and imprecision. Therefore, results close to the 30 ng/mL clinical decision threshold should be interpreted cautiously.^16^,^20^,^21^ Although small systematic differences were observed, limits of agreement remained clinically acceptable at the population level. However, such biases may influence classification outcomes in individual patients with values near clinical decision thresholds, particularly around the 30 ng/mL cutoff used to distinguish vitamin D insufficiency from sufficiency. Correlation analysis further reinforced this alignment, with very strong inter-assay relationships (r = 0.86–0.92) observed across all pairwise comparisons (Figure 2). The highest concordance was noted between the fluorescence-based POC immunoassay and the benchmark automated CLIA platform, while the contemporary automated CLIA platform similarly demonstrated strong alignment with the benchmark system. These findings confirm that both laboratory-based and decentralized immunoassay technologies generate coherent measurement trends across the full clinical range of 25(OH)D concentrations.

Agreement analysis supported these observations. Bland–Altman plots demonstrated modest mean percentage biases (–14.3% to +10.0%) with symmetrical dispersion and no evidence of proportional error (Figure 3). A small number of outliers were observed in the Finecare versus Mindray comparison, where several samples exceeded the upper limits of agreement. These isolated discrepancies may reflect sample-specific matrix effects, variability near assay detection thresholds, or differences in antibody binding characteristics. No consistent demographic or clinical pattern was identified among these samples. However, because additional interference testing and detailed case-level biochemical characterization were not available, the exact cause of these outliers could not be determined.

Additionally, immunoassay-based vitamin D measurements remain inherently susceptible to limitations such as cross-reactivity with vitamin D metabolites, epimer interference, and variability related to vitamin D binding protein concentrations. These factors may contribute to assay-specific variability, particularly in special populations such as pregnant individuals or patients with altered protein-binding states. The study also did not incorporate standardized reference materials such as NIST SRM 972a or LC-MS/MS validation, which may further strengthen future analytical standardization efforts.

Receiver operating characteristic (ROC) analyses demonstrated excellent diagnostic concordance among platforms, with AUC values ranging from 0.961 to 0.985 (Figure 4), indicating highly reliable discrimination between vitamin D insufficiency and sufficiency. These findings were corroborated by categorical performance metrics, including overall percent agreement of 92–93% and Cohen’s κ values of 0.84–0.85 (Table 2), reflecting near-perfect classification agreement. Collectively, the benchmark automated CLIA platform, the contemporary automated CLIA platform, and the fluorescence-based POC immunoassay classified vitamin D status with comparable diagnostic performance.

Specificity and negative predictive value (NPV) were consistently high across all platforms (Table 2). Specificity values of 91–99% indicate strong capacity to rule out vitamin D insufficiency, thereby minimizing unnecessary supplementation. Similarly, high NPVs (89–97%) confirm that individuals categorized as sufficient are highly likely to be truly sufficient. Because insufficiency (<30 ng/mL) was defined as the positive category, these findings reflect reliable avoidance of false-positive insufficiency classifications; an important consideration when minor shifts near clinical thresholds may influence management decisions.

From an implementation perspective, automated CLIA platforms remain well suited for high-throughput laboratory environments requiring standardization, reproducibility, and workflow efficiency. The fluorescence-based POC immunoassay demonstrated laboratory-comparable diagnostic performance while enabling rapid and decentralized assessment, potentially expanding access in outpatient, community, and resource-limited settings. Comparable analytical robustness has been reported for fluorescence-based immunoassay systems across other biomarkers,^22^ further supporting the reliability of this technological approach. The strong alignment of both the contemporary automated CLIA platform and the fluorescence-based POC immunoassay with the benchmark automated CLIA platform suggests that these modalities may be integrated without compromising diagnostic integrity. From an operational perspective, the fluorescence-based POC assay offers substantially shorter turnaround times compared with centralized CLIA workflows, which require batch processing, laboratory infrastructure, and longer reporting timelines. This may provide clinical advantages in outpatient clinics, primary care settings, and resource-limited environments where rapid decision-making is required.

These findings reflect continued progress toward harmonization of vitamin D immunoassays across technological categories. Nevertheless, several limitations warrant consideration. The study was conducted in a single-center cohort and did not stratify analyses by demographic or clinical subgroups that may influence circulating 25(OH)D dynamics or assay behavior. The inclusion of clinically heterogeneous samples represents a strength of the present study, as vitamin D testing is commonly requested in populations such as pregnant individuals, patients with chronic kidney disease, and individuals with bone-related disorders. This approach supports evaluation of assay performance under real-world conditions. Future studies should include adequately powered subgroup analyses in disease-specific cohorts where vitamin D binding protein variation or matrix effects may influence immunoassay performance.^6^,^23^ Future multicenter evaluations incorporating standardized reference materials and international traceability frameworks (eg, VDSP or DEQAS) will be important to further strengthen global assay comparability. The study also did not incorporate standardized reference materials such as NIST SRM 972a or LC-MS/MS validation, which may further strengthen future analytical standardization efforts. An important strength of the present study is the relatively large and clinically diverse sample set. Compared with some published POC vitamin D evaluations, including the AFIAS/Boditech fluorescence immunoassay POCT study that analyzed 56 samples, the present study included 311 serum specimens spanning deficient, insufficient, sufficient, and high 25(OH)D concentrations, thereby improving assessment of agreement across clinically relevant ranges.^24^ In addition, this study focused primarily on inter-assay agreement and diagnostic concordance. Independent precision testing (intra-assay/inter-assay repeatability) of the POC platform was not performed and should be addressed in future validation studies. Because LC-MS/MS was not included, ROC-derived performance should be interpreted as inter-assay diagnostic agreement rather than absolute diagnostic accuracy.

In summary, the three evaluated immunoassay platforms demonstrated excellent analytical agreement, highly consistent diagnostic performance, and strong cross-platform concordance. These results support their practical applicability for routine screening purposes.

## Conclusion

This study evaluated the analytical and diagnostic performance of a contemporary automated CLIA platform and a fluorescence-based point-of-care (POC) immunoassay against a benchmark automated CLIA platform within a clinically diverse cohort. All three systems demonstrated favorable analytical agreement and strong inter-assay concordance for classification of vitamin D insufficiency using clinically relevant thresholds.

The contemporary automated CLIA platform showed performance highly comparable to the benchmark system, confirming reliability for routine laboratory testing. The fluorescence-based POC immunoassay similarly demonstrated favorable agreement with both automated CLIA platforms, supporting its potential utility for rapid and decentralized vitamin D screening in appropriate clinical settings.

Observed inter-platform biases were small and clinically acceptable, suggesting stable cross-platform interpretation even near clinical decision thresholds. Collectively, these findings support the potential utility of modern automated CLIA and fluorescence-based POC immunoassays for routine 25(OH)D evaluation across diverse healthcare settings, while results near clinical decision thresholds should be interpreted cautiously. Continued assay standardization through traceability frameworks such as VDSP and DEQAS will be essential to further improve comparability across platforms and ensure global consistency in vitamin D diagnostics.

## Data Availability

The original contributions presented in this study are included in the article. Further inquiries can be directed to the corresponding author.
